# Current Barriers to Large‐scale Interoperability of Traceability Technology in the Seafood Sector

**DOI:** 10.1111/1750-3841.13796

**Published:** 2017-08-21

**Authors:** Marah J. Hardt, Keith Flett, Colleen J. Howell

**Affiliations:** ^1^ Future of Fish 1201 Alaskan Way, Suite 200 Seattle Wash. 98101 U.S.A

**Keywords:** Interoperability, seafood supply chain, systems change, traceability technology

## Abstract

Interoperability is a critical component of full‐chain digital traceability, but is almost nonexistent in the seafood industry. Using both quantitative and qualitative methodology, this study explores the barriers impeding progress toward large‐scale interoperability among digital traceability systems in the seafood sector from the perspectives of seafood companies, technology vendors, and supply chains as a whole. We highlight lessons from recent research and field work focused on implementing traceability across full supply chains and make some recommendations for next steps in terms of overcoming challenges and scaling current efforts.

## Introduction

Full‐chain digital traceability is the use of electronic records and technology both to track the forward movement of a product through various stages of a supply chain and to trace backward the history of that product, including locations, transformations and applications (Bhatt and others 2016). Today, the nongovernmental organization (NGO) community, national governments, and the seafood sector recognize a need for greater adoption of full‐chain digital traceability to ensure safe, legal, sustainable and accurately labeled seafood products. The European Commission's (EC) catch certification scheme and the U.S. President's IUU (Illegal, Unreported, and Unregulated) Task Force are but two of the most recent examples of this growing awareness (EC 2009; NMFS 2016). The core question among those trying to solve the global overfishing crisis has thus shifted from what needs to be done, to *how* can it be done?

Interoperability—the ability of different information technology systems or software programs to communicate seamlessly for the purpose of exchanging, interpreting and using data (Bhatt and others 2016)—is a critical component of full‐chain digital traceability, but is almost nonexistent in the seafood industry. This study explores the barriers impeding progress towards large‐scale interoperability among digital traceability systems in the seafood sector. We define a barrier as an internal or external factor preventing a particular initiative from gaining scalable traction. Barriers can relate to market conditions, human beliefs and behaviors, cultural norms, or wider issues related to the system in which the barrier exists. Barriers are presumed to be movable, given the right design strategy.

### Current state of seafood traceability

Several studies have attempted to identify barriers that prevent individual companies or subsets of supply chains from adopting traceability technology (Stockdale 2003; Liu and others 2007; Zhang and others 2011; Bosona and Gebresenbet 2013; Chirag 2016; Mattevi and Jones 2016; Siki and others 2012; Visiongain 2014). For the seafood industry some of those barriers include:

- *Lack of awareness of and education* on the need for traceability technology, especially at the full‐chain level;
- *Knowledge gaps* of what full‐chain traceability is and what full‐chain digital traceability does;
- *Poorly demonstrated incentives* for creating buy‐in to the value full‐chain digital traceability can offer;
- *Resource deficiencies*, including funding and capacity issues;
- *Technical issues* with information technology (IT) systems and data management;
- *Logistical hurdles* in the operation of traceability systems; and
- *Scaling issues* in promoting and achieving broader adoption.

These barriers will manifest more or less strongly depending on the structure of the supply chain, supply chain relationship dynamics, and where a company sits within the supply chain (Sterling and others 2015).

In recent decades the seafood industry has experienced an upward trend in seafood supply chain companies adopting and implementing *internal traceability* technologies—those that enable a company to track and preserve information about individual batches or units as those batches or units are processed within a company's facility. Internal traceability solves most of the food safety and recall needs of the food industry. That function alone, however, is not sufficient for product‐level information to be captured, stored and passed along to other trading partners in a manner that provides access to and preserves the integrity of that data so as to maximize traceability benefits across the supply chain, such as by reducing risk and preventing corruption. *External traceability*—the ability to track key data elements (KDEs) and other information about seafood products as they move between trading partners and through the supply chain—must be in place to achieve that higher level of information capture. External traceability hinges on trading partners making commitments to share relevant information with other trading partners, either in one‐up, one‐down fashion or via a cloud‐based system.

External traceability alone, however, does not provide the supply chain transparency, data tracking, or accountability that a company would need in order to ensure that it was not trading in IUU, mislabeled, or fraudulent products—products that, unfortunately, are still prevalent in seafood supply chains. That level of full‐chain digital traceability requires the performance of at least 5 core traceability technology functions including: vessel‐dock capture, product‐data pairing, internal traceability, supply chain visibility, and data verification (see FoF 2016a for a full explanation of these functions). In 2015, Future of Fish (FoF) convened 15 of the leading traceability technology vendors working in the seafood industry and found that while all of these functions were performed by at least 1 of the technology vendors, no single vendor performed all 5 functions.

Thus, 2 types of collaborations are necessary in order for full‐chain digital traceability to succeed. First, multiple supply chain partners must agree to share some level of data. Second, technology vendors must collaborate around supplying services and products within a specific supply chain so that their systems can effectively communicate and interpret this data (for example, interoperability).

### Understanding interoperability

The term interoperability, like traceability, means different things to different people and, thus, deserves explanation. Interoperability is the ability of different information technology systems or software programs to communicate seamlessly for the purpose of exchanging and using data (Bhatt and others 2016). For systems to be truly interoperable, they must have both the capacity to share data using a common data format (*syntactic interoperability*), and the ability to interpret and understand that shared data with common meaning (*semantic interoperability*).

Bhatt and Zhang (2013) highlighted the importance of and need for enabling interoperability for improving the safety and defense of the global food system. They reported on the capabilities of numerous technology solution providers and their inability to interoperate. The EFSA Panel on Biological Hazards (2014) outlined the need to enable interoperability among different datasets to improve outbreak epidemiology, investigation and response. However, even when frameworks and ontologies have been proposed as mechanisms for enabling interoperability (Anonymous 2001; Bhatt and others 2016; Pizzuti and others 2017; Ringsberg 2015), implementation and wide‐scale adoption have been lacking. In fact, interoperability is not just a challenge in the food sector, but rather is an on‐going issue in other industries, such as electronic healthcare records, health informatics, nutrition sciences and dietetics, manufacturing and engineering (Squirrell 1997; O'Sullivan and others 2011; Ayres and Hoggle 2012).

There are currently 3 basic methods for electronically sharing and communicating data.

**Figure 1:**
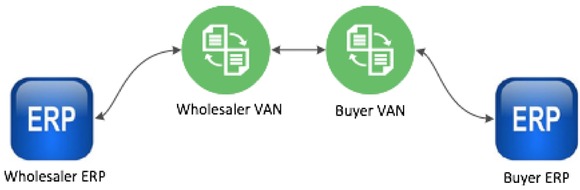
Schematic of a typical EDI data‐sharing system.

The first, Electronic Data Interchange (EDI), shown in Figure 1, is the oldest and most common form and is used often in eCommerce. EDI relies on a text‐based standard for everyday business documents and requires a “middleman” to translate transactions. This middleman, known as a Value Added Network (VAN), is often very expensive, with initial start‐up costs ranging from several tens of thousands to hundreds of thousands of dollars, and transaction charges in the thousands per month (Simmons 2007). Although some modern forms of EDI do allow for data sharing directly between Enterprise Resource Planning (ERP) systems, the method remains restricted to node‐to‐node and does not support full‐chain traceability, nor data sharing among multiple systems.

**Figure 2:**
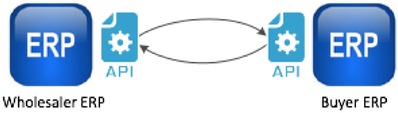
Schematic representing components of API‐based data sharing. In this diagram there are two APIs. The semantic and syntactic definitions are setup to be the same so that communication happens, but this requires programming on both sides.

The second method of data sharing—and one that is gaining traction—is the use of an Application Program Interface (API). Shown in Figure 2, APIs are custom software interfaces that allow 2 distinct systems to communicate electronically. For example, an API for Microsoft Windows helps programmers know how to configure their software to communicate with a Windows platform. Several seafood traceability technology providers have developed custom APIs that allow 1 or more systems to seamlessly share data with those providers; however, these remain limited to the specific systems for which they were built.

While APIs may embed some global standards, they are not based on any end‐to‐end semantic or syntactic interoperability standard and, thus, do not allow data sharing and communication across multiple supply chain nodes. The exception to this is when an API is provided as a service across a supply chain; several technology vendors have developed this functionality, allowing multiple nodes in a supply chain to share data. However, the limitation here is that all electronic traceability systems in that supply chain must conform to the specific API of that single traceability technology vendor. This is not a universal API that would allow sharing across multiple supply chains (unless every seafood supply chain globally was using the exact same traceability service provider).

**Figure 3:**
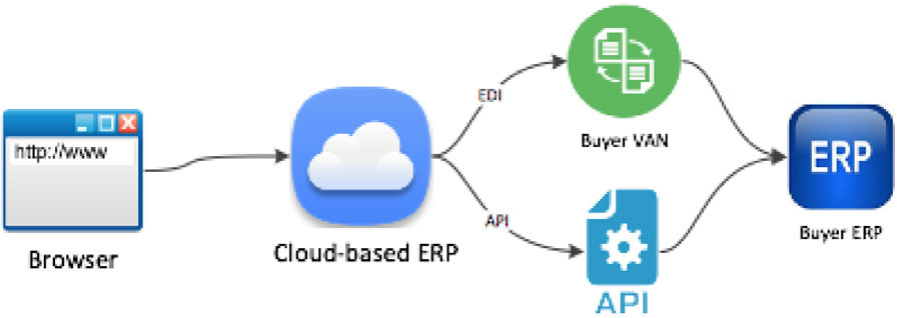
Cloud‐based ERP data sharing system. The seller enters data into their browser, which then connects to the cloud‐based ERP.

The third method of data sharing is a cloud‐based ERP system, which tends to be employed mostly by companies with limited technology capacity. Cloud‐based ERP systems, shown in Figure 3, require data entry via a browser, which is then shared into a cloud‐based database. The information in the database can then be passed, via API or a VAN for EDI, to an ERP system 1 node up or down the supply chain, or to a retailer at the end of the chain. Most often, the host of the cloud‐based data system is not a member of the supply chain, but a third‐party technology provider. While the cloud‐based solution can provide an on‐ramp for electronic data sharing to a company without a more sophisticated system, the additional step of manually inputting data into the browser can be very time consuming, and the system is still limited in terms of where data can be shared, requiring a custom API or a more expensive VAN.

**Figure 4:**
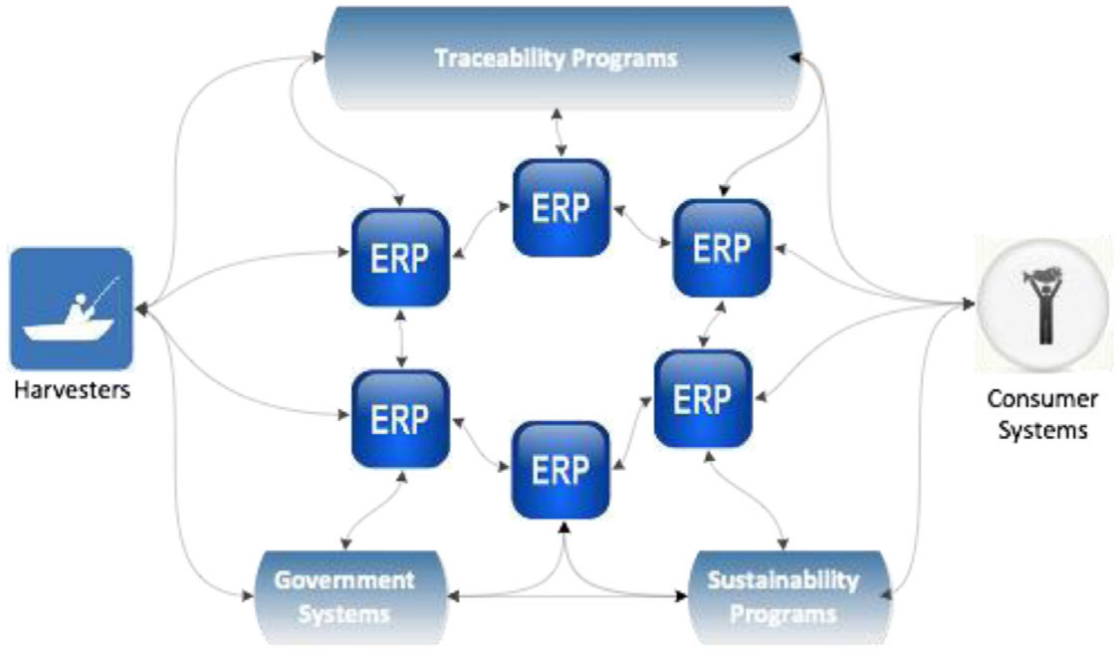
True interoperability, where machine‐to‐machine communication can happen without the need of a translator service or any one service provider, allowing cross‐supply chain and industry data sharing and communication.

In contrast to the 3 methods described above, true interoperability, depicted in Figure 4, allows for unlimited and unfettered machine‐to‐machine data sharing and communication. Two systems can share and effectively interpret data without a translation service. Such true interoperability relies on established standards. Although such standards exist, such as GS1, adoption within the seafood industry sector has been almost nonexistent.

The objectives of this research were to use qualitative methods to:

1. Identify where interoperability exists within the seafood industry and where there are gaps;
2. Identify the challenges impeding interoperability, including nontechnical ones, at both the seafood company and technology vendor levels; and,
3. Highlight existing initiatives working to overcome these challenges.

### Method

Since 2012, FoF, a nonprofit organization, has used a combination of research and design to improve traceability within the global seafood supply chain. In 2015 and 2016, FoF conducted 2 online surveys that solicited information from seafood companies about how they collect and exchange data with their trading partners. FoF developed the questionnaire, which was then shared by NGOs in the sustainable seafood arena who work with industry partners. The first survey specifically targeted companies in North America and the European Union (EU); 56 complete responses were received. A nearly identical survey with an additional question about interoperability was sent to a second sample of seafood companies mostly in North America; 38 complete responses were received. In the case of duplicate responses (that is, both surveys were sent to the same company), the earlier responses were deleted. A copy of the online survey is provided in Table 1. Note that certain questions have been removed for confidentiality purposes. Respondents were not compensated. Our sample is not representative of the entire seafood industry, but rather is biased toward North American companies with some level of commitment to seafood sustainability. Thus, the survey reflects those companies with a higher likelihood of employing electronic data capture, and therefore capable of having their systems interoperate. Also, because we relied on third parties to send out the questionnaires, the response rate is unknown.

**Table 1:** Future of fish seafood traceability technology survey

FOF seafood traceability technology survey (2015)				
Welcome				
The purpose of this survey is to better understand the current methods and technologies seafood companies use to acquire, manage, and track data related to the seafood they buy and sell. We hope to use this research to quantify the degree to which the seafood industry has adopted technology or electronic systems to manage their data, while also understanding the types of systems in use. The survey should take you 5–7 minutes to complete and your answers will remain strictly anonymous. Thank you for your participation. Future of Fish is a nonprofit that works to support and promote business solutions to ocean challenges. The ability to accurately track products (and information about those products) through the supply chain is one way to address some of those challenges, specifically as they relate to IUU fishing, mislabeling, and other forms of seafood fraud. If you have questions or comments please email Future of Fish Research Co‐Director Colleen Howell at chowell@futureoffish.org.				
*Introduction*				
Which of the following best describes your company's business activities? (If your company is vertically integrated, please select all that apply) □ Fisher/farmer □ First receiver □ Primary processer □ Secondary processer □ Broadline distributor □ Wholesale distributor □ Food service □ Retail □ Restaurant (independent) □ Restaurant (chain) □ Importer □ Exporter □ Broker □ Other (please specify)				
Which range best captures your company's total annual sales? □ <$250,000 □ $250,000 ‐ $500,000 □ $500,001 ‐ $1 million □ $1 million ‐ $10 million □ $10 million ‐ $25 million □ $25 million ‐ $50 million □ $50 million ‐ $100 million □ $100 million ‐ $250 million □ >$250 million				
*Traceability*				
Does your company currently use an electronic traceability system? □ Yes □ No				
*Product Information Management*				
Which of the following best describes the system(s) your company uses to collect, store, track, and share information about your products? (Select all that apply) □ Enterprise resource planning (ERP) system □ External cloud‐based traceability system □ Specific accounting, client management, or inventory software (such as warehouse management software, WMS) □ Custom‐built business management system □ Spreadsheet/database software □ Paper‐based system only, no electronic systems or software				

How does your company receive and provide key data about the seafood products you sell?		
	Typically receive data from suppliers via	Typically provide data to consumers via
Purchase orders	□	□
Bill of lading	□	□
Mailed invoices	□	□
Emailed spreadsheets	□	□
Faxes	□	□
Online transaction system	□	□
Paper tickets	□	□
Direct electronic transfer between ERP systems	□	□
Cloud‐based tracebility system	□	□
Point‐of‐sale / Consumer‐facing information	□	□
Other (please specify):	□	□
*No electronic traceability system*		
What is your main reason for not having an electronic data management or traceability system?		
Do you have plans to implement electronic data management or external traceability (which allows data sharing between nodes in the supply chain) in the foreseeable future? □ Yes, within the next year □ Yes, within the next 3 years □ Perhaps, within the next 5 years □ Perhaps, within the next 10 years □ No plans in the foreseeable future		
*Current Data Management or Traceability System*		
Please list the name(s) of the software system(s) you use for electronic data management and traceability: Enterprise resource planning (ERP) system: External cloud‐based traceability system: Specific accounting, client management, or inventory software (such as warehouse management software, WMS): Custom‐built business management system:		

To what extent do you use each of the following data management systems?				
	Only for certain customers	Only for certain products	For nearly all products	For all products
Enterprise resource (ERP) system	□	□	□	□
External cloud‐based traceability system	□	□	□	□
Specific accounting, client management, or inventory software (such as warehouse management software, WMS)	□	□	□	□
Custom‐built business management system	□	□	□	□
*Current traceability system*				
Does your internal data system automatically transfer data to a third‐party electronic system and if so, how? □ No □ Yes, via API □ Yes, via EDI □ Yes, via REST □ Yes, via SOAP □ Yes, via Other (please specify):				
For which of the following do you use your electronic data management and/or traceability technology system(s)? (please check all that apply) □ Capturing product information at the vessel, farm, or dock level (e.g., date of harvest, location of harvest, species name, vessel ID) □ Linking product information to the product itself through a physical identifier (e.g., alphanumeric code, barcode, QR code, or RFID) attached to the product □ Tracking the whereabouts or journey of a seafood product (e.g., where it was harvested or processed, how it was processed, who distributed it, where it was purchased by the consumer) at any point in the supply chain □ Verifying the accuracy of product information □ Viewing company‐level or facility‐level information about the producers, processors, and distributors connected with a seafood product (e.g., company names, locations, health and safety status, certifications, violations, etc.) □ Permission‐based sharing of specific product information with other companies in the seafood supply chain □ Interoperability or integration with other data systems or technologies to allow seamless data‐sharing □ None of these □ Other (please specify):				
*Traceability benefits and challenges*				
You mentioned that your company uses the following system(s) for data management and/or traceability. Briefly, please indicate why your company chose that specific… Enterprise resource planning (ERP) system: External cloud‐based traceability system: Custom‐built business management system: Spreadsheet/database software:				
Does your general customer base know that you use data management or traceability software? □ Yes □ No				
Do you use the data you collect for marketing or branding purposes? □ Yes □ No				
Please list the business benefits your company has realized through your… Enterprise resource planning (ERP) system: External cloud‐based tracebility system: Custom‐built business management system: *Traceability benefits and challenges*				
What are the main challenges your company has encountered with implementing and using your … Enterprise resource planning (ERP) system: External cloud‐based traceability system: Custom‐built business management system:				
***Final Questions***				
*Please provide some general information about yourself and your company*. Company headquarters (City, State/Province, Country):				
In what regions does your company operate? (select all that apply) □ Africa □ Asia □ Central and/or South America □ North America □ Europe □ Oceania				
Which of the following apply to your company? □ MSC Certified Fishery or Chain of Custody (CoC) □ ASC Certified □ BAP Certified □ Involved in Fishery Improvement Projects (FIP) □ GS1 Member □ NFI Member □ FMI Member □ Other (please specify):				
Of which of the following product types does your company sell the most volume? □ Fresh wild finfish □ Fresh wild shellfish □ Fresh farmed finfish □ Fresh farmed shellfish □ Frozen seafood □ Value added products □ Other (please specify):				
Thank you				
Thank you for participating in our survey. If you would like to receive the result of this research, please enter your contact information below. Name: Company: State/Province: Country: Email address: If you have questions or comments please email Future of Fish Research Co‐Director Colleen Howell at chowell@futureoffish.org				

As part of its role in coordinating collaboration among traceability technology vendors, FoF investigated the degree to which interoperability already exists among technology companies working with the seafood industry. Based on FoF's 5 years of work researching traceability technology companies in the seafood sector, and with support from a data architecture consultant, we compiled a list of 37 technology vendors that provide digital traceability software in food systems. Of the 37 companies identified, we were able to secure one‐on‐one phone interviews with 18 vendors based mostly in North America, but also in the EU. All vendors work either primarily in seafood or in the food industry in general, and cover a range of different platforms and services. Vendors discussed the nature of the application/s that they provide, the degree to which their systems are interoperable with other systems, and the costs and benefits they perceive with regard to interoperability.

One objective of the interviews was to create a map of where there was successful interoperability and where there were gaps. As part of the process of gathering information about where, how, and why interoperability was or was not happening in the industry, FoF also collected information about the challenges preventing interoperability from occurring. A discussion guide was used to structure the interviews and included questions such as:

- Have you configured your software so that your customers can electronically exchange data with their customers, suppliers, a government agency, or a third‐party application?
- What challenges have prevented you from integrating with more applications/partnering with other technology companies to integrate?
- When thinking about the seafood industry, what would allow for improved information‐sharing among companies and software systems?

Phone interviews lasted for an average of 75 min. All data collected were provided voluntarily. Respondents received no compensation, but were promised anonymity. A full version of the discussion guide can be obtained by contacting the authors.

The information collected was both qualitative and quantitative. We synthesized the qualitative data by grouping similarly themed responses within the transcripts of interviews and free responses provided in the online surveys. We then looked for patterns that may indicate an overarching barrier, underlying tension, or strategy for progress with respect to advancing and scaling interoperability within the seafood industry. Consultation with data technology experts and leaders of several nonprofit initiatives that are currently conducting traceability pilots, and experience from observations and interviews in the field in conjunction with FoF's numerous traceability studies helped inform the qualitative results.

## Results

### Industry online survey

Of a total of 94 respondents completing at least 1 of the 2 online surveys, 61% reported having some form of an electronic traceability system. Of the 67 MSC‐certified companies in the combined sample, 63% affirmed having electronic traceability, while only 52% of noncertified companies reported employing electronic traceability.

Many companies surveyed are vertically integrated. That is, they are involved with transactions or processes at more than 1 node in the supply chain. For example, of the 25 companies involved with seafood production (fishing or aquaculture), 72% also do some combination of processing (either primary or secondary), distribution, food service, or retail. Roughly 44% of vertically integrated fishing/aquaculture companies have electronic traceability, while only 20% of fishing/aquaculture companies reported electronic traceability systems. Table 2 presents the percentage of companies at each node in the supply chain that employ a traceability system, and shows the degree to which traceability systems are used across vertically integrated companies.

**Table 2:** Use of electronic traceability systems at different positions in the supply chain, and vertical integration in the supply chain

Supply chain position of surveyed company	Traceability system employed	Down‐chain vertical integration (starting at this node)	Vertically integrated with traceability	Nonvertically integrated with traceability
**Production (Fishing or Aquaculture) (*N* = 25)**	40%	72%	44%	20%
**Primary Processing (*N* = 24)**	58%	63%	71%	44%
**Secondary Processing (*N* = 15)**	80%	33%	80%	80%
**Distributor (Broadline or Wholesale) (*N* = 16)**	56%	6%	100%	60%
**Food Service/Restaurant (*N* = 5)**	60%	n/a	n/a	n/a
**Retail (*N* = 3)**	33%	n/a	n/a	n/a
**Import, Export, Broker (*N* = 6)**	67%	n/a	n/a	n/a

Note: In order to prevent double‐counting, vertical integration was defined as being involved with transactions or processes at a downstream node of the supply chain. For example, a processor was considered vertically integrated if it also does wholesale distribution. Processors that were also involved in fishing were considered vertically‐integrated producers and were not counted among processors.

When asked how companies receive data from and provide data to trading partners, most respondents indicated that data are shared using multiple methods, including purchase orders, bills of lading, mailed invoices, emailed spreadsheets, faxes, online transaction systems, and paper tickets. Just 11% of companies receive data via electronic transfer between ERP systems, while 20% said that they provide data via electronic transfer between ERP systems. Less than 10% of surveyed companies receive or provide data with a cloud‐based traceability system.

Companies in the subsample that completed the second survey were asked to indicate the capabilities of their electronic traceability systems (Table 3). The features mirror the 5 core functions identified by FoF, as well as interoperability. Of the 30% of companies reporting to have an internal system to automatically transfer data to a third‐party electronic system, all indicated that they use EDI. Although EDI systems allow for node‐to‐node data exchange, they do not enable full‐chain interoperability or cross‐chain interoperability.

**Table 3:** Capabilities of electronic traceability systems employed in the seafood industry

Traceability System Feature	% of Surveyed Companies (N = 27)
Capturing product information at the vessel, farm, or dock level (for example, date of harvest, location of harvest, species name, vessel ID)	44%
Linking product information to the product itself through a physical identifier (for example, alphanumeric code, barcode, QR code, or RFID) attached to the product	59%
Tracking the whereabouts or journey of a seafood product (for example, where it was harvested or processed, how it was processed, who distributed it, where it was purchased by the consumer) at any point in the supply chain	30%
Verifying the accuracy of product information	22%
Viewing company‐level or facility‐level information about the producers, processors, and distributors connected with a seafood product (for example, company names, locations, health and safety status, certifications, violations, and so forth)	33%
Permission‐based sharing of specific product information with other companies in the seafood supply chain	22%
Interoperability or integration with other data systems or technologies to allow seamless data‐sharing	7%
Internal data system automatically transfers data to a third‐party electronic system	30%

### Phone interviews with traceability technology vendors

Phone interviews with technology vendors revealed diverse capabilities across multiple categories of products and services (Table 4). Of the 18 companies interviewed, only one‐third work exclusively in seafood; most also serve clients in other industries, including meat, produce, and palm oil. Nearly 60% of the vendors interviewed provide cloud‐based software, with an additional 11% of companies providing cloud or premise‐based options.

**Table 4:** Overview of types of technology vendors interviewed about current interoperability capabilities

General traceability function or service category	Number of companies interviewed
Internal traceability (inventory, labelling, shipping and receiving, barcoding, and so forth)	7
External traceability (B2B, B2C, import or export, and so on)	3
Supply chain characterization (transaction monitoring, transparency)	1
Verification and external traceability	1
Vessel‐dock level data capture	3
Warehouse product management	1
Product lifecycle management	1

N = 18

In contrast to the low rate of noted interoperability by seafood companies, approximately half of all technology vendors interviewed stated that they had successfully interoperated with another technology vendor. Of that group, 50% said that they use API as the means of integration. Several companies (21%) used only EDI to integrate, and 14% noted that they used both API and EDI, depending on the system with which they were attempting to interoperate. Other methods of data sharing among vendors included spreadsheets, text, and Extensible Markup Language (XML) based on Electronic Product Code Information Service (EPCIS) and GS1 standards.

### Interoperability mapping

After interviewing and surveying nearly 120 technology companies and seafood supply chain businesses, we found very little evidence of interoperability for entire seafood supply chains that were not either extremely short or already vertically integrated. Thus, the objective to map the state of interoperability within the seafood industry was not met.

### Barriers to interoperability

Comparing results from the online surveys with those from the interviews, we found that 50% of technology providers report having transferred electronic information between seafood trading partners, while only 11% of seafood companies report being involved in such transfers. As big data and information technology play major roles in other sectors, we attempted to identify and understand the barriers to sharing information electronically—both for technology vendors and for seafood companies.

Our process of pattern finding to analyze the qualitative data provided by both interview transcripts and from free responses to the online questionnaires revealed that the barriers to interoperability were largely not technical in nature. That is, interoperability is not necessarily difficult from a system‐talking‐to‐system standpoint. Rather, interoperability is stalled because of human psychology, business culture and systemic issues that no single company or supply chain can solve independently. These findings build off those of Bhatt and others (2016) and others who noted that failings in information flow stemmed not just from technological deficiencies but often from weaknesses in intra‐and inter‐business relationships.

The remainder of this paper summarizes the findings of our qualitative research into the factors that seem to be impeding scaled interoperability.

## Discussion

Our discussion focuses on the barriers to interoperability from the perspectives of seafood companies, technology vendors, and supply chains as a whole. Additional factors, such as value chain type, have also been shown to influence the kinds of barriers preventing interoperability (Bhatt and others 2016). These factors will be important to consider in terms of how seafood companies and technology vendors may need to prioritize strategies for addressing the challenges noted here. To assist with this, we also describe several underlying tensions or counteracting forces that often create inertia or perpetuate the status quo (that is, no interoperability). And, we offer lessons from recent research and field work focused on implementing traceability across full supply chains, and make some recommendations for next steps in terms of overcoming barriers and scaling current efforts.

### Barriers to seafood companies pursuing interoperability

Before interoperability can be implemented, it must be recognized. The barriers described below reflect factors that seem to be preventing companies from becoming aware of interoperability and recognizing its value. These barriers emerged as themes based on a process of pattern‐finding to analyze the qualitative data from the online questionnaires.

#### An industry culture of competition, not collaboration

Interoperability requires a level of inter‐business collaboration that is unprecedented in the seafood industry. Seafood businesses work on small margins and with a self‐protective skepticism around the trustworthiness of competitors and trading partners. They guard their supply chains and sourcing practices closely, fearing that leaked information could damage business and reduce profits. The idea of interoperability—where specific product‐level data are shared machine‐to‐machine all along the supply chain—is not only perceived as too risky, but is also antithetical to current industry culture.

#### Discounted value of interoperability

Traceability itself is relatively new to the industry, and the concept of interoperability is not even on the radar for many seafood companies. Thus, while the value of interoperability may be clear to those who understand its potential, most seafood executives are either unaware, or downplay the benefits because they are too hypothetical, long‐term, or uncertain. The gains companies tend to realize through their traceability systems have to do with internal efficiencies, maintaining contracts with high‐value customers, or marketing. Some find it difficult to imagine the complete benefits, especially supply‐chain level benefits that interoperability makes possible through robust, end‐to‐end traceability. Others see the benefits as purely social (such as improvements in human rights across the industry as a whole) and, thus, not within their individual business interests. Making data capture and sustainability information relevant to seafood companies is a challenge, especially when consumer demand for detailed data is perceived as relatively low.

### Barriers to seafood companies implementing interoperability

Once seafood companies are aware of the value of interoperability, they face new challenges related to implementation. The barriers described below emerged as themes based on a process of pattern finding to analyze the qualitative data from the online questionnaires.

#### Scarce resources are already fully tapped

Interoperability requires both capital and human resources. Even when a seafood company's leadership sees the potential value of integrating with other systems, the cost of implementation can be prohibitively high. This is especially true for custom ERP systems and legacy systems. Interoperability implementation can require significant attention from IT staff (where such staff exist), many of whom already have more work than they can handle. Seafood companies without IT staff have no choice but to hire an IT consultant to do the work, or to pay the premium that most ERP systems charge for custom integrations. Adding to the financial burden, interoperability implementation and the related back‐end code updates can take the entire system offline for weeks. Most companies cannot afford that level of disruption to their operations.

### Barriers to technology vendors pursuing interoperability

In addition to the factors impeding adoption of interoperability among seafood companies, technology vendors face their own barriers when it comes to integrating with other platforms. The following barriers emerged as themes based on a process of pattern finding to analyze the qualitative data from the interviews with technology vendors.

#### Perceived risks outweigh uncertain benefits

While the risks of interoperability perceived by seafood companies concern privacy and security, traceability technology vendors see interoperability as potentially jeopardizing their business futures. They cannot afford the costs to both their bottom lines and their reputations of attempting to interoperate with another vendor—especially a young, unproven start‐up, as many traceability vendors still are—without guarantee of success. Some traceability companies have made false claims about the services that they provide and, thus, vendors interested in integrating with other vendors have to do their own vetting process before moving forward with partnerships. Interoperability among vendors occurs most often when integration is requested from their clients (that is, seafood companies), which is relatively rare for the reasons described earlier.

### Barriers to technology vendors interoperating

#### System incompatibility

The manifold electronic data systems used in the seafood industry presents a significant challenge to interoperability. In general, integrating with an ERP system is more straightforward than integrating with a cloud‐based traceability system. However, older systems, custom‐built systems, and platforms built on obsolete operating systems (Windows 95, for example) can pose incompatibilities that are not rectifiable and, as a result, interoperability partnerships are abandoned. In many cases, interoperability requires system‐to‐system customization, which when trying to link an entire supply chain may mean designing custom integrations with several very different systems, each built on their own platforms.

### Barriers to seafood supply chains interoperating

In the case of both seafood companies and technology vendors, interoperability will only succeed to the extent that all players in the supply chain are willing and active participants. The barriers described below reflect factors that impede entire supply chains from interoperating. These barriers emerged as themes based on a process of pattern‐finding to analyze the qualitative data from both the online questionnaires and the interviews with traceability vendors.

#### A chain is only as strong as its weakest link

Interoperability is not simply a decision made between 2 trading partners; all supply chain actors must comply and demonstrate at least a minimum level of commitment. Thus, supply chains that include businesses that lack traceability cannot interoperate; supply chains that include businesses that cannot (for cost or technical reasons) or refuse to integrate their systems even though all other trading partners are on board cannot interoperate. Meanwhile, supply chains that include businesses using traceability technologies that are inferior with respect to data handling or data security, may not realize the full benefits of interoperability, or may decide that interoperating is too risky.

#### Lack of consistent data standards

Interoperability requires sets of standardized data formats and data fields that all systems can follow. Although certain standards (for example, GS1 and EDI) exist, most seafood companies are not members of such schemes, namely due to cost and lack of demand from their customers for such standards. Given that most companies have their own internal product SKUs, even if systems are made to speak the same language (syntactic interoperability), they must also be able to exchange data in a meaningful way (semantic interoperability). Thus, if a seafood company or technology system is GS1 compliant, for example, but the data received from the supplier are not formatted correctly, the information cannot be passed along. The absence of universal product codes for the hundreds of thousands of different combinations of species, product types, product forms, product weights, and so forth passing through seafood company databases may be one of the most formidable barriers to interoperability. Further complicating the situation, some major buyers have their own “flavors” of EDI, forcing customization even within a “standard.”

### Opportunities for moving forward

This research highlights the range of technological, financial, logistical, and cultural barriers to interoperability currently facing companies and technology vendors working within the seafood supply chain. As with any system‐level problem, tackling these barriers in order to scale interoperability requires a comprehensive strategy—one that is beyond the capacity of any single company, government, or organization to achieve. However, individual efforts working in concert can provide the multi‐pronged approach necessary to initiate forward progress.

The following initiatives are examples of ongoing work to address particular barriers to interoperability in the seafood supply chain. Some of these efforts are more closely coordinated than others. Based on our findings, continued dialogue and knowledge sharing may be fruitful as these projects progress in order to accelerate interoperability and ultimately, adoption of digital traceability systems across seafood supply chains.

#### The seafood traceability technology architecture and rollout strategy

This project, led by the Institute of Food Technologists’ Global Food Traceability Center (GFTC) is intended to address the growing need for a global, secure, interoperable seafood traceability system by designing a common technology architecture. Details of this work are presented in articles by Bhatt and Gooch (2017), Bhatt and others (2017), and Gooch and others (2017) on pages 22 and 45 of this supplement.

### Barrier addressed: *lack of consistent data standards*

#### Financing full‐chain traceability

FoF is leading efforts to develop novel ways of financing the implementation and long‐term maintenance of full‐chain traceability. Through this effort we hope to help alleviate some of the burden that interoperability brings, especially to seafood producers and processors. By reinventing the traditional transaction model, this initiative seeks to unlock the full value that accurate, shared data provides to seafood companies, governments, and NGOs and move digital traceability from something currently funded by foundations to an investment made by industry.

### Barriers addressed: *an industry culture of competition, not collaboration*; *scarce resources are already fully tapped*

#### Catch documentation and traceability architecture

With the GFTC, FoF is currently under contract with the U.S. Agency for International Development's (USAID) Ocean's and Fisheries Partnership Program to design a technology architecture for interoperable communications between traceability vendors, supply chain members, governments, and others to effectively communicate information that meets the KDE and privacy needs of fishery stakeholders (USAID 2017). The result of this work will be a published and vetted architecture that allows interoperable web services to effectively and efficiently communicate among data technologies.

### Barrier addressed: *lack of consistent data standards, system incompatibility*

## Conclusions

If you build it, they may come; but then again, they might not.

When asked, the majority of those involved in the seafood industry—from seafood companies to NGOs to government officials—tend to assume that the biggest hurdle to interoperability is technical in nature. That is, technology vendors still have not done the work to make their systems compatible. Yet, research shows that this is not the case. The technical ability to join 2 data systems has long been available. It is the scaling of that process that remains stalled.

Generally speaking, traceability technology providers see value in interoperating, both in terms of services to their clients, for growing their customer bases, and for competitive advantage. Yet, demand for interoperability is currently lagging.

Today, technology companies have 2 choices: either pursue interoperability despite lack of customer demand in hopes that existing and future customers will realize the benefits and eventually come on board; or wait for demand to hit critical mass and then spring to action. Either decision requires a leap of faith: the former involves significant upfront investment with no guarantee of returns while the latter risks missing out on the potential advantage of being among the interoperability pioneers. Accelerating buy‐in to the value of interoperability (and traceability) requires efforts to align supply chains and effectively strategize solutions to the cultural elements and relationship dynamics in the system. The good news is that these efforts have already begun.

### Mitigating risk, embracing opportunity

A primary goal of the current interoperability initiatives outlined here, and highlighted in this supplement, is to help mitigate risk for technology companies, and to support the seafood industry as it transitions to digital traceability solutions.

The GFTC has identified several key principles that were critical for successful scaling of interoperability as it occurred in multiple industries. These are presented in detail in the article—

*Implementing Interoperability in the Seafood Industry: Learning from Experiences in Other Sectors*—by Bhatt and others (2017) on page 22 of this supplement. These principles provide additional guidelines for how we might most effectively engage industry partners and technology vendors to achieve true interoperability across the seafood industry.

Current efforts to interoperate are stuck at the small scale. But the barriers impeding progress can be moved—especially now that they have been identified in a more holistic fashion. The subsequent articles in this Supplement provide further insight and opportunities for how we can leverage these insights for continued progress.
